# Patients’ and providers’ perspectives on non-urgent egg freezing decision-making: a thematic analysis

**DOI:** 10.1186/s12905-023-02189-3

**Published:** 2023-02-08

**Authors:** Leah Drost, E. Shirin Dason, Jinglan Han, Tanya Doshi, Adena Scheer, Ellen M. Greenblatt, Claire A. Jones

**Affiliations:** 1 Department of Obstetrics and Gynecology, Mount Sinai Hospital, Sinai Health System, Toronto, ON M5G 1X5 Canada; 2 Department of Obstetrics & Gynecology, Temerty Faculty of Medicine, University of Toronto, 12Th Floor, 123 Edward St, Toronto, ON M5G 1E2 Canada; 3 Department of Obstetrics &, Gynecology University of Saskatchewan, Saskatoon, SK S7N 0W8 Canada; 4 Department of General Surgery, St. Michaels Hospital, Unity Health Network, Toronto, ON M5B 1W8 Canada

**Keywords:** Fertility preservation, Cryopreservation, Reproductive behaviour, Qualitative research

## Abstract

**Background:**

The decision to undergo non-urgent egg freezing (EF) is complex for patients and providers supporting them. Though prior studies have explored patient perspectives, no study has also included the separate perspectives of providers.

**Methods:**

This qualitative study involved semi-structured individual interviews exploring the decision to undergo EF. Participants included patients considering EF at one academic fertility clinic and providers who counsel patients about EF from across Canada. Data analysis was accomplished using thematic analysis. Data saturation was met after interviewing 13 providers and 12 patients.

**Findings:**

Four themes were identified and explored, illuminating ways in which patients and providers navigate decision-making around EF: (1) patients viewed EF as a ‘back-up plan’ for delaying the decision about whether to have children, while providers were hesitant to present EF in this way given the uncertainty of success; (2) providers viewed ovarian reserve testing as essential while patients believed it unnecessarily complicated the decision; (3) patients and providers cited a need for change in broader societal attitudes regarding EF since social stigma was a significant barrier to decision-making; and (4) commonality and peer support were desired by patients to assist in their decision, although some providers were hesitant to recommend this to patients.

**Conclusions:**

In conclusion, the decision to undergo EF is complex and individual patient values play a significant role. In some areas, there is disconnect between providers and patients in their views on how to navigate EF decision-making, and these should be addressed in discussions between providers and patients to improve shared decision-making.

**Supplementary Information:**

The online version contains supplementary material available at 10.1186/s12905-023-02189-3.

## Background

Due to the educational and professional advancements of women as well as improvements in contraceptive methods, the age of childbearing has increased over time; women are generally starting families in their late twenties and thirties [1–3]. However, it is well-known that infertility increases with age. As a result, non-urgent egg freezing (i.e. egg freezing without an urgent oncological or other medical necessity), which allows the preservation of fertility at a younger age, has become an appealing option for women who wish to delay childbearing [3].

Although non-urgent egg freezing (EF) is becoming increasingly common [3, 4], it remains controversial for non-oncologic situations due to the potential ethical issues involved [5] as well the lack of good data on live birth rates [3]. Previous studies have explored reasons why women choose to undergo non-urgent EF. Lack of a partner or having a partner who is not ready for parenthood is described as the most common reason for pursuing EF [6–8], as well as the sense of running out of time to have children biologically [9]. Gaining a sense of control over reproductive potential and/or a romantic future has also been described in the literature as a reason to pursue EF [10, 11].

Making the decision to undergo EF can be difficult for patients. Many women who pursue EF describe feeling stressed about needing to decide whether to have children when they are not yet ready to make this decision, and relief in being able to defer this decision through EF, giving them a greater sense of control over their reproductive planning [12]. Some women consider pursuing EF to avoid feelings of regret in the future should they experience involuntary childlessness, while others are concerned about decisional regret—particularly if they are not successful in achieving a live birth after pursuing EF [8, 9, 12]. Patients have described feeling embarrassment and shame for being in a position in life where they are considering freezing their eggs [9] which may complicate decision-making. Furthermore, the cost of EF is a potential barrier to pursuing EF; in Canada, the average cost per patient for an EF treatment cycle and storage can range from CAD$5,000–$10,000 not including medication costs and is clinic-dependent. Additionally, costs may be higher for some patients if they require more than one cycle to achieve a sufficient number of eggs.

Previous studies exploring decision-making in the context of EF have focused primarily on the patient perspective. Given the complexity of this decision, healthcare providers play an important role in counselling women who are considering EF via a shared decision-making process. This process involves a healthcare provider helping a patient consider their values in a medically complex decision. Shared decision-making is a method which is becoming increasingly common across the field of obstetrics and gynecology [13]. The main objective of this qualitative study was to assess the complexity of the decision to undergo EF for non-medical indications, taking into consideration the perspectives of patients making this decision, and the separate perspectives of healthcare providers supporting these patients via a shared decision-making lens.

## Methods

### Ethics approval and informed consent

Approval was obtained prior to initiation of the study from the Mount Sinai Hospital Research Ethics Board (# 17-0001-E). All methods were performed in accordance with the Declaration of Helsinki. All study participants provided initial and ongoing informed consent.

### Recruitment and data collection

Participants were part of two populations selected by purposive and convenience sampling, and these included both: (1) providers across Canada who are involved in counselling women about egg freezing as a reproductive option, including reproductive endocrinology and infertility (REI) physicians, nurse practitioners, and reproductive counsellors; and (2) English-speaking patients over the age of 18 who had attended an academic fertility clinic (Mount Sinai Fertility: Toronto, Ontario) for EF consultation. Patients with a diagnosis of cancer or other medical illness requiring urgent treatment impacting fertility were excluded. Patients were recruited via posters in the clinic or were introduced to the study by their attending physician. A research assistant then asked the patient if they would like to be contacted for the study. Providers were recruited via email invitation.

Data collection consisted of individual interviews with each participant using a semi-structured interview guide, conducted in person or over the telephone over a period of 2 years. Interviews were conducted by study investigators L.D., S.D., and J.H. as well as by research assistant M.S. Investigators who interviewed participants were all healthcare professionals involved in the care of the study population, or professional student learners. There was no relationship between the investigators and the participants.

### Interview guides

Two similar interview guides were designed by the authors, based on the Ottawa Decision Support Framework (ODSF) [14] and adapted for each population (Additional file 1: Appendix A). The ODSF is a standardized framework for assessing difficult decisions where there are multiple values-based options and aims to help guide researchers and practitioners in assessing people’s decisional needs and providing decisional support. The guides consisted of open-ended and closed-ended questions about the decision to undergo EF or not. Topics explored with both populations included the options and alternatives when considering EF, factors that influence their decision-making, decisional supports available, and barriers they face in the process; providers were also asked about the support that they provide to patients facing this decision and any challenges they face with providing this care. Patient advisors were not involved in the conception of study design or interview guide.

The interview guide was considered fluid, and concepts were freely explored as they were brought up by participants. The interview guide was modified along the course of the interviews to explore concepts and themes more fully as they were introduced by participants. Interviews were recorded and transcribed verbatim by L.D., S.D., J.H. and T.D, and a second study investigator checked each transcription for accuracy. Qualitative research concepts were integrated into data collection and analysis, including emergent findings, reflexivity of the researcher and ongoing analysis.

### Data analysis

Exploration of decisional needs, barriers and a potential decisional aid are reported in a separate paper; the current paper focused on thematic analysis of open responses. Thematic analysis of the data included development of codes, concepts, categories and theories [15] about the decision to undergo EF. Analysis was performed simultaneously with interviews in an iterative process, and interviews were conducted until thematic saturation was met [16]. Two study investigators (L.D. and S.D.) independently coded interviews using NVivo12, and an audit trail of annotations and coding was kept to ensure reliability of data. Similarities and discrepancies in coding, themes and concepts were discussed by L.D., S.D., C.J., E.G., and T.D. to come to an agreement.

## Findings

### Characteristics of participants

Twenty-three patients were approached about the study, of whom 14 agreed to participate and were interviewed (60.9%). Reasons to decline to participate were not provided. Two interviewed patients were excluded from data analysis as they were considering EF for medical indications. The majority of patients were age 35–39, and were well-educated with all having at least an undergraduate degree (Table 1). All patients had decided to proceed with (*n* = 9), or were seriously considering proceeding with EF (*n* = 3). Responses were similar amongst patient groups.

**Table 1:** Demographics

Demographic	*n* (%)
*Patients*	*N* = 12
Age (years)	
30–35	3 (25.0%)
35–39	8 (66.7%)
Not asked	1 (8.3%)
Education	
University/college undergraduate	5 (41.6%)
University graduate	4 (33.3%)
Medical degree	1 (8.3%)
Medical and graduate degree	1 (8.3%)
PhD	1 (8.3%)
*Providers*	*N* = 13
Years in practice	
<5	4 (30.8%)
5–10	4 (30.8%)
10–20	5 (38.5%)
Gender	
Female	7 (53.8%)
Male	6 (46.2%)
Practice role, discipline	
Physician, GREI	9 (69.2%)
Psychologist/psychotherapist, Fertility	2 (15.4%)
Nurse practitioner, Fertility	1 (7.7%)
Social worker, Fertility	1 (7.7%)
Location	
Ontario	6 (46.2%)
Nova Scotia	3 (23.1%)
Alberta	2 (15.4%)
British Columbia	2 (15.4%)

Twenty-eight providers from across Canada were approached about the study, of whom 13 agreed to participate and were interviewed (46.4%). Those who did not participate did not respond to the recruitment email and thus no reason was provided for declining the invitation. Years in practice varied among providers, and the majority were physicians specializing in REI across Canada (Table 1).

### Themes

**Fig. 1:**
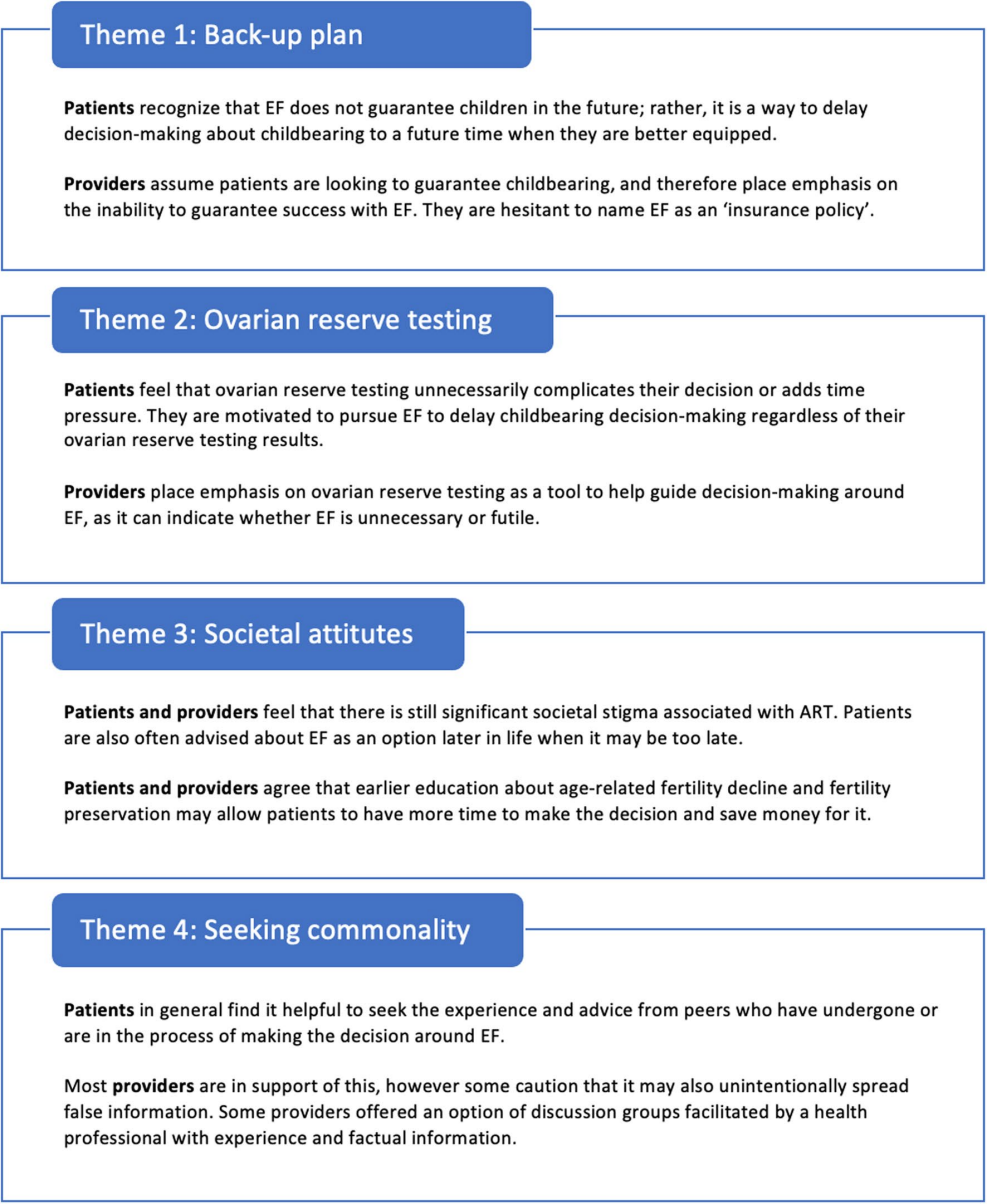
Themes. *ART* assisted reproductive technology, *EF* egg freezing

Thematic analyses of open responses revealed four broad themes about the decision to undergo EF: the concept of EF as a “back-up plan”; the impact of ovarian reserve testing results on decision-making; the need for change in societal attitudes around fertility preservation and assisted reproduction; and the desire amongst patients to find shared experiences and commonality amongst their peers who are facing or have faced the same decision (Fig. 1).

#### Theme 1: Egg freezing as a “back-up plan”

The concept of EF as a “back-up plan” was frequently raised by both patients and providers with very different interpretations of this term. Patients generally revealed that they considered EF as a way to delay the decision about whether they wanted to have children at all to a future time when they would be better equipped or ready to think about such a decision. Overall, patients had accepted the fact that EF does not guarantee a future child, and several patients did explicitly recognize that future success with their frozen eggs was uncertain. However, patients still maintained that even without guaranteed success, it was still a viable option for them: “*Even though there’s nothing guaranteed, it’s a chance and for some people like me, a chance is still a chance and that’s that*.” At the forefront of patient’s minds were the ideas of advancing their career and finding the right partner before deciding whether they wanted children, and there was often still some uncertainty about having children in general. One patient shared, “*I would love to have kids, but at one point I was thinking is it really a big deal if I don’t have kids. But then again, I always come back to the idea that it’s like an insurance, a backup plan. Even if like I decide not to have kids, like that would reduce some of the stress of getting older, not having a partner, and-and helps me not to think that my biological clock is ticking, so it’s kind of like a back-up plan, assurance for me*.”

Providers largely assumed that patients were looking at EF as a way to guarantee having a child in the future. They were apprehensive that patients did not put enough thought into the uncertainty of successfully achieving a pregnancy from the use of frozen eggs in the future. One provider suggested: “*The process does give you… a bit of peace, like it might kind of psychosocially allow you to kind of relax a little bit knowing that you’ve got at least something frozen. And then the downside of that is that it might make you feel complacent, like ‘Hey I’ve got those eggs, I don’t really have to worry about my aging and fertility anymore’ which isn’t exactly true because those eggs may not work*.” This demonstrated a disconnect between the way providers approached counselling and the decision that patients felt they were making.

This theme was further illustrated by the different ways in which EF was compared to the concept of an insurance policy by both patients and providers. Patients ultimately highlighted the idea of never needing to use the “insurance policy”, while providers highlighted that if patients did need to use the “insurance policy”, it would not be as reliable a “pay out” as other common “insurance policies”. The differing perception of the role of EF as insurance is highlighted by one patient: “*this is just another insurance policy… and if you decide not to, by 40, 42, whatever it might be, then you don’t. And yes, it’s a lot of money, but no different than, you know, paying home insurance and never needing to use it*.” Echoing this, one provider (a reproductive counsellor) described the sense of relief that patients have when they know egg freezing is an option for them: “*They feel less pressure about having to make any sort of decisions around childbearing right now, so like I said before, it’s like an insurance policy for them*”. In contrast, some providers emphasized the endpoint of most insurance policies as being the reason for getting insurance, deliberating that perhaps “insurance” is a misnomer when compared to other common insurance policies: “*As much as… people describe it sort of as insurance, it’s not really the case. Insurance is something where if you crash your car when you’re driving and you have insurance, your insurance company will provide you with a new vehicle. But if you freeze your eggs, it’s not insurance that you’re going to have children in the future, it just reduces the chance that you won’t not have children in the future if you want to*.” Another provider reiterated this, saying, “*it’s not a guarantee, we don’t call it fertility insurance, we call it sort of hedging your bets*”. Patients did not seem as concerned about this idea that the “insurance” of EF may not pay out in achieving a live birth should it need to be used in the future.

#### Theme 2: The role of ovarian reserve testing results in decision-making

Ovarian reserve testing was frequently raised as an important consideration by providers, but was rarely raised by patients as being a factor that influences the decision to undergo EF. Nearly half of providers (*n* = 6) emphasized the importance of ovarian reserve testing (i.e. anti-Müllerian Hormone, AMH) to guide patient’s decision-making as it has a role in predicting success in achieving live birth from EF.

Providers described how they interpret ovarian reserve testing to help counsel the patient about whether it makes sense to pursue EF or not. Providers commented that they would consider people with a low AMH to be poor candidates to pursue EF, because of having a low predicted number of eggs retrieved per EF cycle which would result in a low chance of achieving a future pregnancy through EF. However, several providers also stated that they considered people with low ovarian reserve testing to also have a lower chance of achieving pregnancy spontaneously later in life, and would be more likely to go through menopause earlier and therefore might still want to consider EF for this reason, despite the overall poor prognosis. This poor prognosis in the setting of diminished ovarian reserve makes counseling about EF challenging for providers since they worry that women with a low AMH who choose to do EF will unrealistically rely on EF as a good chance of achieving pregnancy in the future. Similarly, providers commented that patients who have a high AMH are both good candidates for spontaneously achieving pregnancy later in life, and have more time to make the decision about EF. In general, ovarian reserve testing seemed to be essential to decision-making around EF for providers counseling patients, because of the focus that providers place on the success of achieving a live birth through EF: “*I think just having a complete picture of the puzzle… knowing more about their actual numbers, like ovarian reserve and measures of ovarian reserve is helpful*.” Some providers also commented that they needed the testing to help them decide whether they should play a more active role in decision-making by emphasizing the futility of treatment, while other providers stated that the results of testing simply led to more personal discomfort for themselves if patients decided to go forward despite being counselled that EF would be unlikely to lead to successful outcomes (in the case of a low AMH) or unnecessary (in the case of a high AMH).

In contrast, most patients did not bring up the results of initial ovarian reserve testing as being important to the decision to undergo EF. When prompted about the role of ovarian reserve testing, patients commented that they thought that the purpose of testing was to assess the ability to conceive now, which they felt was irrelevant to their decision as they were looking to delay decisions about conception to a future time. Similarly, even if their ovarian reserve testing was low, patients still wanted to preserve whatever fertility they had left, even if their chance of successfully achieving a pregnancy through EF was low. They were not willing to pursue childbearing now or to delay treatment until a future time. Those few patients who did bring up initial testing commented that this only complicated their choice or added time pressure as they were motivated to pursue egg freezing regardless of what the testing showed; they also described confusion about the implications of the test results. One patient shared, “*I’d say the first doctor I saw, when they gave me my numbers it was kind of like, okay you can’t do it, and then I was like okay if they’re saying I can’t do it then I have limited time to actually do this if I’m going to try…I didn’t feel any pressure from the doctors because they were telling me not to do it, but yeah*.” Furthermore, a few patients described ovarian reserve testing as a negative influence on their decision to pursue EF: “*Honestly it was quite an emotional roller coaster, because I was also told that I probably shouldn’t do it. [Interviewer: By who?] A doctor… because my numbers weren’t very high… When I was told not to I was very strongly discouraged and really upset, and I actually went to see another doctor. And they were a little bit more encouraging, but for a good few months I was really just, you know, put off the idea*.” This same patient later brought up how although the testing results did not affect her ultimate decision as she ended up pursuing EF, it did affect her choice of provider as she opted to work with a physician who was more encouraging despite her test results. Patients make the decision of whether to pursue EF and preserve their fertility regardless of how successful EF is likely to be in achieving a future pregnancy, and therefore ovarian reserve testing does not factor into their decision.

#### Theme 3: Call for change in broader societal attitudes regarding egg freezing

Providers and patients alike brought up the need for normalization of fertility treatment/support. This was illustrated by the existing stigma of assisted reproductive technology (ART), as well as the desire to have this information brought up earlier in life to provide patients with more time to consider egg freezing. Some providers and patients shared that the stigma of ART and the possibility of being a single parent can make patients feel ashamed to share this decision with others close to them, limiting their ability to seek support from family or friends. Although providers generally indicated that stigma around these issues was improving over time, it still remained a barrier facing patients considering EF. Furthermore, the stigma associated with talking about fertility issues in general was identified as a barrier for women learning about fertility preservation options, thereby diminishing the time available to make this often time-sensitive decision. One patient commented on this and called for a change in how we talk about fertility in society: “I* think because we don’t really talk about it in the world, or in our world at least, no one realizes like there’s stuff you can do to help yourself, but you have to be young enough to do it. We always realize it like when we’re old and trying to create families at that point and then it might be too late for a lot of people. So, I think as a culture if we actually were more open around fertility things, miscarriages and fertility issues in general… I think a huge bunch of [women] would choose to do egg freezing in their mid to late twenties so then at least they know that when they are ready to have a family that option is readily available to them*.”

Echoing this sentiment, many patients and several providers mentioned that a majority of patients only learned later in life that fertility declines with age and that fertility preservation is an option. Upon learning this information later in life, individuals considering fertility preservation are faced with an enormous time pressure to make a decision which was perceived as a significant barrier to effective decision-making. As one patient shared, “*I was thinking about when I was 25 and I wish it was promoted a little bit more. I wish it was talked about, I wish we were told this, in school about reproduction and really what the truth is about life because then we could have made better-informed decisions… Getting into the world of fertility, I can't believe what a world it is and how many challenges people do have and I think it needs to be discussed so that people can see that there are options there for them, and they can get whatever they want*.” Both providers and patients acknowledged that earlier education about age-related fertility decline and the potential for preservation of one’s fertility might lead to women having more time both to make the decision and to save money for it. As one patient shared, “*If someone had said to me in my twenties even, like save a little money and go do this in a few years, I would have really appreciated that*.” Recognizing a missed opportunity earlier in life, one provider commented: “*If the decision could be made-or the consideration of doing this could be made when people have a better chance for success, that would be a huge plus. Because that’s a barrier for proceeding, because most clinics won’t do it past 38… Like involving primary care more, so that instead of preventing pregnancy their whole life they actually talk about family planning as well before it’s too late*.” When asked about the best model of delivery of this type of educational information, providers’ and patients’ opinions ranged from implementing fertility-related content into middle- and high-school curricula, to encouraging primary care providers (both family physicians and general gynecologists) to bring up fertility decline and available options with patients in their late twenties to early thirties.

#### Theme 4: Seeking commonality and peer support

Many patients sought commonality in experience amongst friends and online sources such as blogs and discussion boards. They were keen to find others who had undergone or were in the process of deciding about EF, in order to normalize their situation and to provide more specific advice about the process and what to expect from personal experience. One patient shared, “*I think I had all the resources really at my disposal… what helped me was sort of reading these blogs online of women who have done this… and even [talking] to a couple of friends of mine who are going through something similar—hearing that direct experience is helpful. It's a little bit more specific about sort of the overall process and what like the day-to-day kind of is*.” Another patient who did not personally partake in discussion groups remarked on how, if they had been available to her, they may have helped her with her decision: “*I think the discussion groups… might have been helpful to see how other people were thinking about it, and what kinds of questions they're asking themselves and how they're answering them. Just because it gives you other ideas that maybe haven't occurred to you*.”

However, some providers felt that online sources or discussion groups could be detrimental—that there was a high proportion of skewed perceptions found on online sources, either positive or negative, which they ultimately felt was not helpful in decision-making. When asked if discussion groups would be helpful, one provider shared: “*I’m not sure. Sometimes it makes me think it’s something called ‘pooled ignorance’, where they just talk about what they think is best, and then they’re not experts in the area, so… Maybe for support, but in the decision-making, I’m not sure*.” Other providers thought that because each patient’s situation and likelihood of success was so unique, seeking commonality amongst peers would not be helpful. One provider remarked that they would not recommend discussion groups, “*because [patients] all have their unique situations with their age and their ovarian reserve tests*.” Still other providers were in support of peer support in the form of a discussion group, as long as it was facilitated by a health professional with experience and factual information.

## Discussion

Our study is unique in that we separately explored the perspectives of patients considering egg freezing, as well as the providers who support them in this decision, which enabled us to identify four important themes—some with differing perspectives between patients and providers. These themes included the concept of EF as a “back-up plan”, the role of ovarian reserve testing in decision-making, the broader societal attitudes around fertility preservation, and the desire for peer support. Each theme highlights potential areas of improvement in the counseling of patients, in order to better support decision-making for patients considering EF.

The concept of EF as a “back-up plan” has been identified in previous research [17]. Our results highlight that there is a disconnect between provider and patient views on this matter. Providers generally assume that patients are definitely planning to have children in the future, and thus place emphasis on the inability to guarantee success with EF. However, patients in our study, and in previous studies, are not under the false impression that egg freezing is a guarantee [8]. Rather, they are looking to EF as a means to delay the decision about whether to have children or not until they are ready to consider this, for example at a time when their career or relationship status is more amenable to parenting. This is further highlighted by the well-known concept that most women do not return to use their frozen eggs, so success in live birth outcomes is not necessarily their ultimate goal; rather, it is about preservation of some form of fertility.

A novel finding in our study was the difference in opinion between providers and patients regarding ovarian reserve testing as a way to guide decision-making. The evidence on the utility of ovarian reserve testing is unclear; although markers of ovarian reserve, including AMH, have been shown to be an effective way to predict the number of retrievable eggs [3], several studies have found that it does not necessarily correlate with success in achieving a pregnancy or live birth [18, 19]. The ACOG recommends that AMH should not generally be performed or used to counsel patients who are not infertile [20], which includes the patients who generally seek out EF. As such, it is interesting that nearly half of providers emphasized that this was an important part of their counselling of patients. Further research could explore the explicit views and patterns of practice of providers regarding the role of AMH in EF. Patients in our study did not identify AMH as a component which helped their decision-making. Rather, some specifically mentioned that it unnecessarily complicated their decision as they were not sure how to interpret the findings and what it meant for them. Further, contrary to the current evidence around AMH, patients misunderstood the purpose of AMH, and believed that it quantified their ability to produce a live birth at the current moment. Our study suggests that ovarian reserve testing should only be undertaken for EF cycle planning in order to help a patient understand how many cycles to plan on (and financially invest in) rather than as a measure of the futility or necessity of treatment, or as a way to predict chance of live birth. Patients should be properly educated on the utility and limitations of AMH, and a patient’s goals of treatment should be explored and prioritized prior to obtaining ovarian reserve testing. How testing results might impact treatment should be discussed explicitly with patients prior to encouraging them to complete tests, especially if the test may add to the financial burden of treatment.

Our study also supported previous findings about the broader social attitudes regarding fertility preservation and EF in particular [9, 21], demonstrating that there is a lack of overall social understanding and awareness of EF as an option for women to delay childbearing; our findings highlight that there is a wish amongst patients pursuing this option that this were more well-established and accepted in society. Furthermore, the gap in education about age-related fertility decline and reproductive options was another theme that surfaced in our study which echoes previous research [7]. The majority of patients as well as several providers in our study mentioned the need for better and earlier education about fertility decline. This finding, together with the previous research, highlights the need for more providers, such as family physicians and general gynecologists, to bring up age-related fertility decline and the consideration of egg freezing for those patients wishing to delay childbearing—in the same way contraception is brought up as part of general family planning. Similarly, age-related fertility decline should be included in school curricula and public health campaigns. This will allow women to become better informed about their reproductive health and options, and could potentially lead to better accessibility of EF. Furthermore, consideration should be given to the role of society in providing supports to allow women greater flexibility in family planning while pursuing their careers, rather than constraining women to hold off on childbearing until the time is right for their careers.

The fourth and final theme identified in our study highlighted the desire of many patients to seek out advice and support from peers, particularly from those who have also undergone or are making a decision about EF. Although each person considering EF has their own unique needs and values, and requires individualized counselling [22], patients may find it helpful to additionally have access to support/discussion groups or forums comprised of other individuals who are facing a similar decision. In one study, women who had already undergone EF suggested that support groups facilitated through in-vitro fertilization clinics would have been helpful [23]. Such groups or forums, in person or online, could provide additional practical information or considerations—for example, what to expect during the process, or what additional resources others have found helpful. Some of the providers in our study pointed out that it may be prudent to consider guidance by a health or social work professional who is knowledgeable about the topic and could appropriately guide discussions.

A strength of our study was the involvement of both providers and patients interviewed separately, as this allowed a broader look into the decision-making process and more clarity on the disparities of opinions. Furthermore, a diverse group of providers was interviewed, both by geography (across Canada) and discipline. Finally, the semi-structured interviews allowed the investigators to explore concepts freely as they were brought forward by participants. Limitations of our study included the lack of representation from patients who decided not to undergo EF; though we invited both patients who chose to undergo egg freezing and those who chose against it, we were only able to recruit and complete interviews with patients who chose to go through with EF. Further studies should aim to capture the perspectives of women who consider EF but choose not to proceed with it. Additionally, patients were only recruited from a single academic, urban fertility clinic and were highly educated, therefore the views of the patients in this study are not representative of the entire population of women who consider EF across Canada. Furthermore, certain demographic and cultural factors such as race/ethnicity, religion, and family beliefs were not collected, which could limit contextualization of the findings; future studies should seek to collect this data. Finally, although we reached data saturation amongst the populations we interviewed, we cannot be sure that we would not gain additional information from further interviews.

## Conclusions

In conclusion, our study identified four broad themes which each highlighted the complexity of the decision about whether to undergo EF—for patients, as well as for providers counselling them. In some areas, including the notion of EF as a “back-up plan” and the use of ovarian reserve testing, there is a disconnect between provider and patient views which may contribute to the well-documented difficulty of the EF decision-making process, and should be discussed to better understand patients’ goals and values. Furthermore, there is a need for earlier education and information about age-related fertility decline and fertility preservation so that patients may be better equipped financially and emotionally to make this decision. Finally, patients may desire peer support and experience in assisting with decision-making and should be considered. In addressing each of these themes, we may improve patient care and experience in decision-making around EF.

## Supplementary Information

**Additional file 1. Appendix 1.** Interview Guides (Patient and Practitioner).

## Data Availability

The data underlying this article cannot be shared publicly due to the privacy of the individuals that participated in the study. The data will be shared on reasonable request to the corresponding author.

## Abbreviations

AMH: Anti-Müllerian hormone
ART: Assisted reproductive technology
EF: Egg freezing
