# Supplementary material for: Patients’ and providers’ perspectives on non-urgent egg freezing decision-making: a thematic analysis
Source: BMC Womens Health. 2023 Feb 8;23:49. doi: 10.1186/s12905-023-02189-3 (PMC9906951; doi:10.1186/s12905-023-02189-3)
Supplement: Supplementary file 1 — Additional file 1. Appendix 1. Interview Guides (Patient and Practitioner). [file 12905_2023_2189_MOESM1_ESM.docx]

**Appendix A.1: Interview Guide (Patient)**

May I confirm that you have made a decision about whether or not to proceed with elective egg freezing?

1. At this time, what do you think are the most important decisions facing people who want to delay childbearing?

2. We’re going to focus on the decision of whether or not to undergo elective egg freezing:

3. Thinking about this decision, what were the options that you had?

4. What do you see as the main advantages/benefits and disadvantages/risks of the options?

| Option | Advantages/Benefits | Disadvantages/risks |
| --- | --- | --- |
| 1. |  |  |
|  |  |  |
|  |  |  |
| 2. |  |  |
|  |  |  |
|  |  |  |
| 3. |  |  |
|  |  |  |
|  |  |  |

| 5. Let’s talk about the challenges with making this decision about elective egg freezing. How did you feel when making this decision? | Did you feel:   - Unsure about what to do - Worried what could go wrong - Distressed or upset - Constantly thinking about the decision - Wavering between choices or changing your mind - Delaying the decision - Questioning what is important to you - Feeling physically stressed (tense muscles, racing heartbeat, difficulty sleeping) - Other |
| --- | --- |
| 6. What things made the decision difficult for you? | Were you:   - Lacking information about options, benefits, or risks - Lacking information on chances of benefits and harms - Confused from information overload - Unclear about what is important to you - Feeling unsupported in decision making - Feeling pressure from others - Lacking motivation or not feeling ready to make a decision - Lacking the ability or skill to make a decision - Other |

| 7. Who else was involved in making this decision with you? | Do they usually:   - Make the decision - Share the decision - Provide support or advice for you to make decision on their own - Other |
| --- | --- |
| 8. How do you usually go about making such a decision? | Do you:   - Get information on options - Get information on chances of benefits and risks - Consider the personal importance of the benefits and risks - Get information on how others go about deciding - Get support from others - Find ways to handle pressure - Other |

9. What would have helped you make this decision?

9A. At what point(s) in the process did you feel you needed help with making the decision?

- Prior to first visit at Fertility Clinic
- During first visit at Fertility Clinic
- After first visit at Fertility Clinic
- Other (specify)
- No help needed

10. Was there anything that got in the way of making this decision?

11. Was there anything else that would have helped overcome these barriers to decision making?

12. I will list possible ways to help some people with a decision. Which ones do you think may have been useful to you?

| - Counseling from health practitioner | If YES, specify what types |
| --- | --- |
| - Discussion groups of people facing same decision | If YES, specify what type of organization or group |
| - Information materials | If YES, specify content:   - Options - Costs of treatment - Benefits - Risks - Probabilities of benefits/risks - Help considering personal importance of benefits versus risks - Guidance in the steps of deliberation and communication - Other, specify |
|  | If YES, specify format   - Booklets, pamphlets - Internet – website - App - Videos/DVD - Other, specify |

13. Was there anything else that would have supported you better in decision making?

CHARACTERISTICS OF PATIENT:

14. Age category (guestimate)

- < 30
- 30-35
- 35-39
- 40+

15. What is the highest grade of level of education you completed?

- High school or less
- Some college/university
- University undergraduate degree
- University graduate degree (Masters or PhD)

16. Decision made

- Proceed with social egg freezing
- Do not proceed

**Appendix A.2: Interview Guide (Health Practitioner)**

1. What decisions do patients wanting to delay childbearing have to make in your practice? Probe: Do they make decisions about electively freezing eggs?

2. Let’s focus on one particular decision: the decision about whether to pursue elective egg freezing.

3. What do you see as the main options patients have in making this decision?

4. What do you see as the main advantages/benefits and disadvantages/risks of the options?

| Option | Advantages/Benefits | Disadvantages/risks |
| --- | --- | --- |
| 1. |  |  |
|  |  |  |
|  |  |  |
| 2. |  |  |
|  |  |  |
|  |  |  |
| 3. |  |  |
|  |  |  |
|  |  |  |

| 5. Let’s talk about the difficulty patients have making this decision about elective egg freezing. How do patients feel when making this decision? |  | Do you think patients feel:   - Unsure about what to do? - Worried what could go wrong - Distressed or upset - Constantly thinking about the decision - Wavering between choices or changing their mind - Delaying the decision - Questioning what is important to them - Feeling physically stressed (tense muscles, racing heartbeat, difficulty sleeping) - Other |
| --- | --- | --- |
| 6. What makes the decision difficult for patients? |  | Are patients:   - Lacking information about options, benefits, risks - Lacking information on chances of benefits and harms - Confused from information overload - Unclear about what is important to them - Feeling unsupported in decision making - Feeling pressure from others - Lacking motivation or not feeling ready to make a decision - Lacking the ability or skill to make a decision - Worried about cost |

| 7. What is your usual role in making this decision? | Do you usually:   - Make the decision for the patient - Share the decision with the patient - Provide support or advice for patients to make decision on their own - Other |
| --- | --- |

8. What factors make it difficult for you to support your patients’ decision making?

9. What factors make it easier for you to support your patients’ decision making?

| 10. Who else besides yourself and the patient is usually involved in making this decision? | - Spouse - Family - Friend - Another Health care provider - Other (specify) |
| --- | --- |
| 11. What is their usual role in making the decision (i.e. person mentioned above) | Do they usually:   - Makes the decision for the patient - Share the decision with the patient - Provide support or advice for patients to make the decision on their own - Don’t know - Other (specify) |
| 12. How do patients usually go about making such a decision? | Do they:   - Get information on options - Get information on changes of benefits and risks - Consider personal importance of benefits and risks - Get information on how others go about deciding - Get support from others - Find ways to handle pressure |

13. What would help patients to make this decision?

13A. At what point(s) in the process do you feel decision support should be offered to best help patients with making the decision?

- Prior to their first visit at Fertility Clinic
- During first visit at Fertility Clinic
- After first visit at Fertility Clinic
- Other (specify)
- No help needed

14. What hinders patients (get in the way of) making this decision?

15. I will list possible ways to help some people with a decision. Which ones do you think might be useful to your patients for this decision?

| - Counseling from health practitioner | If YES, specify what types |
| --- | --- |
| - Discussion groups of people facing same decision | If YES, specify what type of organization or group |
| - Information materials | If YES, specify content:   - Options - Benefits - Risks - Probabilities of benefits/risks - Costs of treatment - Help considering personal importance of benefits versus risks - Guidance in the steps of deliberation and communication - Other, specify |
|  | If YES, specify format   - Booklets, pamphlets - Internet – website - App - Videos/DVD - Other, specify |

16. Is there anything else that would help overcome barriers in decision making?

17. Is there anything else that would help you to do a better job supporting your patients’ decision making?

CHARACTERISTICS OF PRACTITIONER:

18. Number of years in practice

- <5 years
- 5-10 years
- 10-20 years
- Over 20 years

19. Sex

- Male
- Female

20. Practice discipline and role
